# Long-term all-sites cancer mortality time trends in Ohio, USA, 1970–2001: differences by race, gender and age

**DOI:** 10.1186/1471-2407-5-136

**Published:** 2005-10-20

**Authors:** Jerzy E Tyczynski, Hans J Berkel

**Affiliations:** 1 Cancer Prevention Institute, Dayton, Ohio, USA

## Abstract

**Background:**

There were significant changes in cancer mortality in the USA over the last several decades, in the whole country and in particular states. However, no in depth analysis has been published so far, dealing with changes in mortality time trends in the state of Ohio. Since the state of Ohio belongs to the states of relatively high level of all-sites mortality in both males and females, it is of interest to analyze recent changes in mortality rates, as well as to compare them with the situation in the rest of the USA. The main aim of this study was to analyze, describe and interpret all-sites cancer mortality time trends in the population of the State of Ohio.

**Methods:**

Cancer mortality data by age, sex, race and year for the period 1970–2001 were obtained from the Surveillance Research Program of the National Cancer Institute SEER*Stat software. A joinpoint regression methodology was used to provide estimated annual percentage changes (EAPCs) and to detect points in time where significant changes in the trends occurred.

**Results:**

In both, males and females mortality rates were higher in blacks compared with whites. The difference was bigger in males (39.9%) than in women (23.3%). Mortality rates in Ohio are generally higher than average USA rates – an overall difference was 7.5% in men in 1997–2001, and 6.1% in women. All-sites mortality trends in Ohio and in the whole USA are similar. However, in general, mortality rates in Ohio remained elevated compared with the USA rates throughout the entire analyzed period. The exceptions are the rates in young and middle-aged African Americans.

**Conclusion:**

Although direction of time trends in Ohio are similar in Ohio and the whole US, Ohio still have cancer mortality rates higher than the US average. In addition, there is a significant discrepancy between white and black population of Ohio in all-sites mortality level, with disadvantage for Blacks. To diminish disparities in cancer mortality between African Americans and white inhabitants of Ohio efforts should be focused on increasing knowledge of black people regarding healthy lifestyle and behavioral risk factors, but also on diminishing socioeconomic differences, and last but not least, on better access to medical care.

## Background

Mortality from cancer is an indicator of the effectiveness of cancer control efforts in a given population, and, thus, is an important measure from the public health point of view. Declining cancer mortality rates may indicate that cancer prevention activities have been successfully designed and implemented, may also indicate favorable results of the implementation of improved therapeutic procedures, as well as successful early detection programs. On the other hand, increases in mortality rates can indicate failures in controlling cancer risk factors and/or an appearance of new ones. Hence, the analysis of cancer mortality time trends and patterns in a given population can be helpful in assessing successes, failures and future need in cancer control programs.

There were significant changes in cancer mortality in the USA over the last several decades. These changes were described many times, however, dealing mainly with mortality in the USA as a whole [1-5]. Much less has been published on cancer mortality time trends in particular sub-populations, at the state or county levels [6-8]. In particular, no in depth analysis has been published so far, dealing with changes in mortality time trends in the state of Ohio. Since the state of Ohio belongs to the states with relatively high level of total cancer mortality in both males and females, it is of interest to analyze recent changes in mortality rates, as well as to compare them with the situation in the rest of the USA.

There are several methods of assessing changes in cancer mortality trends over time. The simplest way is based on visual inspection of rates, while more sophisticated methods are based on statistical modeling of observed data (e.g. age-period-cohort modeling). For the purpose of this study we decided to employ a joinpoint regression analysis (a non-linear regression modeling known as piece-wise or segmented regression). This approach was chosen to allow for detecting points in time where significant changes in the direction of trend occurred, as well as to assess average percentage changes in mortality rates.

The main goal of this paper is to analyze and discuss changes in total cancer mortality in the state of Ohio, and to compare this to the national USA patterns, and discuss possible reasons for existing differences both within Ohio, and between Ohio and the USA as a whole.

## Methods

Cancer mortality data for all sites (ICD-9 codes 140–208) for the state of Ohio and for all states combined were obtained from the NCHS via Surveillance Research Program, National Cancer Institute SEER*Stat software – version 5.2.2 [9]. The data were available for the period 1970–2001. Corresponding population data, by age, sex, race and year, were extracted from the same source.

Age-standardized mortality rates (ASRs) were calculated for each calendar year, for all ages combined and for the following age groups 20–44, 45–64, and 65 and over (for each gender separately, for all races combined and for Whites and African Americans separately). The World Standard Population was used for age-adjustment [10]. To assess the most recent differences between sub-populations (by gender, age and race) average age-standardized mortality rates were also calculated for the last 5-year period (years 1997–2001 combined). Percentage differences for the period 1997–2001 between rates were calculated for blacks and whites, for particular age categories, and between Ohio and the USA.

A joinpoint regression was fitted to provide estimated annual percentage change (EAPC) and to detect points in time where significant changes in the trends occur [11,12]. A Joinpoint software version 2.6 was used [11]. For each EAPC estimate we also calculated the corresponding 95% confidence interval (95%CI). A maximum number of 3 joinpoints was allowed for estimations.

The joinpoint regression model describes continuous changes in rates and uses the grid-search method to fit the regression function with unknown joinpoints. In this model, the annual age-adjusted rates over a given period of time are examined and the points in time when the direction of the trends changes significantly are detected [12]. Thus, joinpoint is a useful way to summarize trends in cancer rates, and it allows one to assess recent changes in trend.

## Results

### General pattern of cancer mortality in Ohio

**Figure 1:**
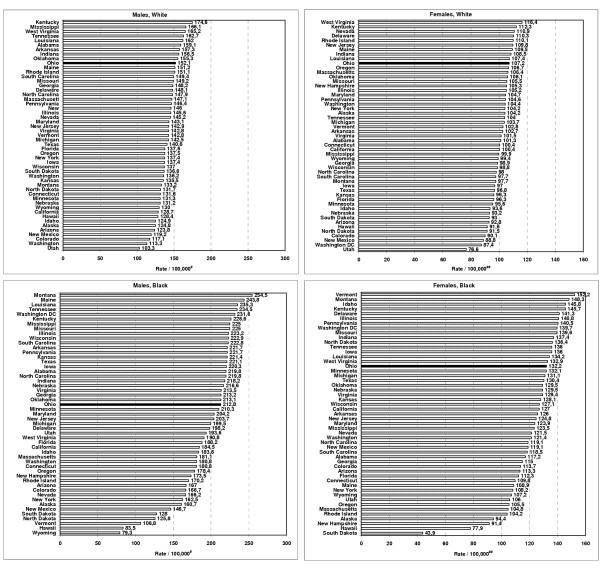
Cancer mortality in the USA, 1997–2001, by state, gender and race # – Age-adjusted mortality rates (World Standard Population).

There were an average of 12,800 cancer deaths per year in men in Ohio in the period 1997–2001 (11,380 in white men and 1,400 in African Americans). In women, the average number of deaths per year in that period was 12,060 (10,800 and 1,260 in white and black populations respectively). An average age-adjusted mortality rate was 156.4/100,000 in males and 109.0/100,000 in females. In both, males and females mortality rates are higher in black populations compared with whites (Figure 1). The difference is bigger in males (39.9%) than in females (the difference of 23.3%). The biggest difference in rates between black and white populations was observed in males in the age group 45–64 year (53.7%), whilst the lowest difference was observed in elderly women (aged 65 and over) – 19.8% (Table 1). Mortality rates in Ohio are generally higher than average USA rates – an overall difference in men was 7.5% in 1997–2001, and 6.1% in women (Table 1). Also in particular age categories overall (in all races combined) mortality rates in Ohio were higher compared to the USA average – the biggest difference was noted in elderly men (aged 65 and more) – 8.7% (Table 1). The most frequently recorded cancer site was lung – in both males and females, and in both whites and blacks (Table 2). In white males the second most frequent site was colorectal followed by prostate, while in black males the second most frequent site was prostate (Table 2). In women, in both whites and blacks, lung was followed by breast and colorectal cancers (Table 2).

### Changes in mortality in time – overall

**Figure 2:**
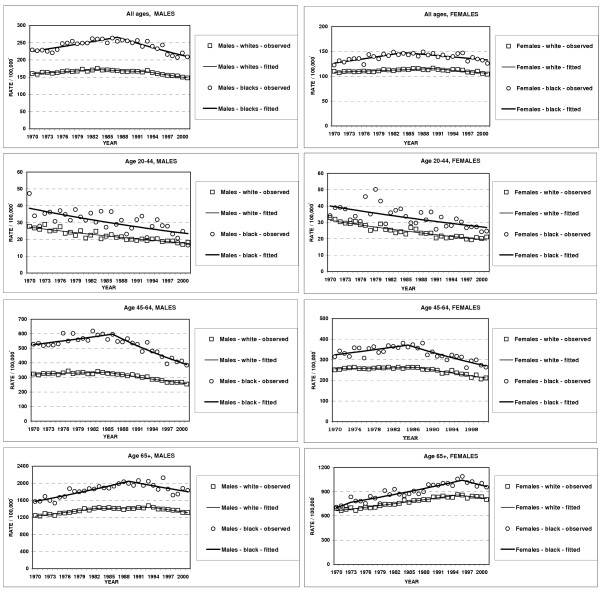
Cancer mortality time trends, all sites, Ohio, 1970–2001, by race # – Age-adjusted mortality rates (World Standard Population).

Results of the joinpoint analysis of mortality time trends are shown in Table 1 and in Figure 2. For all ages combined in males, after a moderate increase of 0.7% per year until 1982, a significant decline in mortality occurred later on, reaching -1.5% per year after 1993 (Table 1). In women, mortality rates were going up until the end of 1980s, a significant, but moderate increase of 0.4% per year, after which a decline occurred with -0.8% per year (Table 1).

In young adults (aged 20–44 years) a permanent decline in mortality was observed in both males and females. Also, the EAPCs for both genders were similar (-1.4% and -1.5% per year in men and women respectively) (Table 1). In middle-aged men and women (45–64 years of age) a pattern of changes in time was also similar: after small increase by 0.3% per year in 1970–1985, a decline of rates occurred since 1986 (the EAPC for males was -1.9% and for females -1.5%) (Table 1). In older men (aged 65 and more) an increase of mortality was observed in the 1970s (by 1.2% per year), followed by plateau in the 1980s and beginning of the 1990s, and then followed by decrease by -1.3% since 1994 (Table 1). In elderly women an increase of mortality by 1.1% per year was observed until 1994, and then followed by significant decline in mortality (EAPC -0.9%) (Table 1).

### Changes in mortality by race

There are some differences in time trends development between white and black populations. In men, all-ages mortality was increasing faster in blacks than in whites, and decline in African Americans commenced later than in whites. In women, the situation was opposite – mortality started to decline earlier in African American women (in 1984) than in white women (1990) (Table 1). In young adults, permanent decline in mortality was noted in both genders, regardless the race. Also the EAPCs were similar in both races in young people. In middle-aged white men plateau in mortality was observed until the mid of 1980s, while in black middle-aged men an increase by 0.8% per year was observed in the same time. Since the mid 1980s a decline in mortality was observed in both races, however higher EAPC was noted in African Americans (-2.8%) compared with whites (-1.6%) (Table 1). In middle-aged women, decline in mortality commenced earlier in blacks (1985) than in whites (1987) and was more pronounced in the former (-2.0% vs -1.5%). In the older men (65+) decline in mortality began in 1989 in African Americans and in 1994 in white men, and was similar in both races (-1.0% and -1.2%) (Table 1). In women aged 65 years and more decline in mortality has been observed since the mid of the 1990s and approximately two-fold faster in African American women (-1.7% per year) compared with white women (-0.9%) (Table 1).

### Time trends in Ohio vs. whole USA

**Figure 3:**
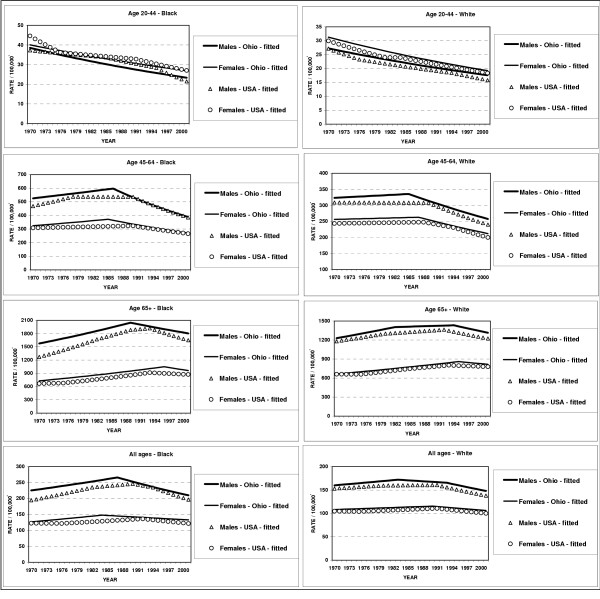
Cancer mortality time trends, all sites, Ohio vs USA, 1970–2001, by race # – Age-adjusted mortality rates (World Standard Population).

All-sites mortality trends in Ohio and in the whole USA are similar, however, in general mortality rates in Ohio remained elevated compared with the USA rates throughout the entire analyzed period. The exceptions are the rates in young and middle-aged African Americans. In middle-aged African Americans rates were at the similar level in Ohio and in the USA in the second half of the 1990s and the beginning of the 2000s (Table 1, Figure 3), in both males and females. In young African Americans (20–44 years of age) mortality rates in 1997–2001 were lower in Ohio than in the whole USA (Table 1).

Despite the differences in the absolute level of mortality rates in Ohio and the USA, trends are, in general, similar in directions. However, in the most recent periods the overall rates (for all-ages and all races combined) have been declining faster in the USA than in Ohio (Table 1), and this difference applied mainly to black populations. This phenomenon is also visible in young adults (20–44) where mortality seems to decline faster in the USA than in Ohio, especially in African Americans of both genders (Table 1). In contrast, mortality rates are declining faster in Ohio in the oldest age category, especially in women (in both races).

## Discussion

The main aim of this study was to analyze, describe and interpret all-sites cancer mortality time trends in the population of Ohio State. We also attempted to compare the results for Ohio with those obtained for the whole USA. Some brief analyses of mortality time trends in Ohio were published before [13,14]. However, they dealt with all-ages time trends only, and did not use any statistical modeling to be applied to the observed data. In our analysis we have applied Joinpoint regression approach (called also stepwise approach) to examine data and to quantify observed changes. It is, to our knowledge, the first analysis of this type done for the Ohio population.

The population of Ohio is heterogeneous – according to the 2000 US Census, 85% of the population was white, 11% were African Americans, and 4% were other race. The heterogeneity is also visible in cancer mortality rates in Ohio. In all age categories and in both genders mortality rates in African Americans were elevated compared with whites. A similar phenomenon has been observed in the whole USA population. However, these differences are not identical in Ohio and in the USA, especially in young adults. In young people the difference in mortality between blacks and whites in Ohio is approximately 25%, while in the whole USA it reaches nearly 50%. Also in middle-aged males and females, as well as in elderly men differences between races are smaller in Ohio compared with the whole country. The only exception is the oldest age category in women, where mortality in Ohio exceeds that of the USA. The phenomenon of elevated cancer mortality in African Americans compared to non-Hispanic whites is well known in the literature [15]. It is also well recognized that people in lower socio-economic groups of the society tend to have higher cancer mortality rates than wealthy people. It was shown, among others, for breast cancer in black and white American women [16]. It is, thus, not surprising that also in Ohio cancer mortality is higher among African Americans compared with whites.

The question is why the difference between the two races is generally lower in Ohio than in the USA. It has been suggested by Bach and colleagues that the differences in survival (and consequently in mortality) are not because of biological factors, but more likely caused by other factors such as differences in treatment, stage of disease at the diagnosis, and co-morbidity (influence of other diseases) [17]. One of the possible explanations for the difference between Ohio and the USA as a whole is the access to the health care system for African Americans, measured by the percentage of people without health insurance. It has been pointed out by Prothrow-Stith and colleagues that lack of health insurance contributes to increased morbidity and mortality from cancer [18]. For example, in the year 2001 in the whole USA 19.0% of blacks had no health insurance, while in Ohio 16.2%. In the same year the proportion of uninsured whites was in the USA and in Ohio virtually the same, 10.0% and 9.7% respectively [19,20]. Our analysis showed that in young and middle-aged individuals, although overall cancer mortality (for all races combined) was higher in Ohio than in the USA, this phenomenon was not present in the black population of that age (Table 1). Moreover, in young adult African Americans (aged 20–44) all-sites cancer mortality was lower in Ohio than in the whole country. Also in all-ages mortality in men the difference between Ohio and the USA was two-fold higher for all races combined and for whites compared with blacks.

The difference between African Americans and white population in health insurance may also influence the difference in cancer mortality between the two sub-populations. In Ohio, black people constitute more than one fifth (22.2%) of all uninsured individuals, while they constitute only 11% of the whole population [21].

Similarly to the USA as a whole, cancer mortality rates have been decreasing also in Ohio. Favorable trends has been observed in the 1990s and the beginning of the 2000s in both males and females, for all ages combined, as well as in all analyzed age categories. However, while mortality was declining throughout the whole period among young people, decrease appeared in the mid of the 1980s in middle-aged individuals, and only in the mid of the 1990s in the oldest age group.

Although cancer mortality rates in Ohio are higher in African Americans compared with whites, recent trends in rates are favorable in both groups. All-sites cancer mortality is mostly influenced by those sites, which make the biggest proportion of cancer deaths. Hence, changes in mortality from the most common sites determine changes in all-sites mortality trends. In Ohio males, in both whites and blacks, three most common cancer sites (lung, prostate, and large bowel) constitute more than 50% of all cancer deaths. An analysis of lung cancer mortality in Ohio showed declining trends in both races [22]. In females, similarly to men, three the most frequent sites (lung, breast and large bowel) form over 50% of all cancers. Lung cancer mortality has been plateauing in women in both races in the 1990s [21].

There is a need for more detailed analysis of time trends for particular cancer sites and/or groups of sites, to be able to define these areas where intervention would be most desired. The most striking phenomenon observed in all-sites cancer mortality among Ohio inhabitants is a gap between black and white populations, with strongly unfavorable patterns among Blacks. It seems that in order to diminish disparities in cancer mortality between African Americans and white inhabitants of Ohio efforts should be focused on increasing knowledge of black people regarding healthy lifestyle and behavioral risk factors, but also on diminishing socioeconomic differences, and last but not least, on better access to medical care [23].

## Pre-publication history

The pre-publication history for this paper can be accessed here:



## Figures and Tables

**Table 1:** All-sites cancer mortality time trends, 1970–2001, Ohio and USA, by gender, age and race

**Sex, age, race**	**Rate 1997–2001^##^**	**Difference (%) Ohio vs. USA**	**Difference (%) Blacks vs Whites**	**Trend 1**		**Trend 2**		**Trend 3**		**Trend 4**	
				**Years**	**EAPC^#^**	**Years**	**EAPC^#^**	**Years**	**EAPC^#^**	**Years**	**EAPC^#^**

***All ages***											
**Males, OHIO**	**156.4**	**7.5**		**1970–1982**	**0.7a**	**1983–1992**	**-0.4a**	**1993–2001**	**-1.5a**		
**Males, OHIO, white**	**152.1**	**7.3**	**39.9**	**1970–1982**	**0.5a**	**1983–1992**	**-0.4a**	**1993–2001**	**-1.4a**		
**Males, OHIO, black**	**212.8**	**3.1**		**1970–1986**	**1.0a**	**1987–2001**	**-1.7a**				
*Males, USA*	*145.5*	-		*1970–1979*	*0.5a*	*1980–1989*	*0.1*	*1990–1992*	*-0.8a*	*1993–2001*	*-1.8a*
*Males, USA, white*	*141.7*	-	**45.7**	*1970–1979*	*0.4a*	*1980–1990*	*0.1*	*1991–2001*	*-1.5a*		
*Males, USA, black*	*206.4*	-		*1970–1981*	*1.6a*	*1982–1989*	*0.6a*	*1990–1993*	*-1.2a*	*1994–2001*	*-2.5a*
											
**Females, OHIO**	**109.0**	**6.1**		**1970–1989**	**0.4a**	**1990–2001**	**-0.8a**				
**Females, OHIO, white**	**107.2**	**5.4**	**23.3**	**1970–1989**	**0.3a**	**1990–2001**	**-0.8a**				
**Females, OHIO, black**	**132.2**	**5.9**		**1970–1983**	**1.1a**	**1984–2001**	**-0.6a**				
*Females, USA*	*102.7*	-		*1970–1974*	*-0.2*	*1975–1990*	*0.4a*	*1991–2001*	*-1.0a*		
*Females, USA, white*	*101.7*	-	**22.7**	*1970–1974*	*-0.2*	*1975–1990*	*0.4a*	*1991–2001*	*-1.0a*		
*Females, USA, black*	*124.8*	-		*1970–1975*	*-0.1*	*1976–1991*	*0.7a*	*1992–2001*	*-1.3a*		
											
***Age 20–44***											
**Males, OHIO**	**18.4**	**7.0**		**1970–2001**	**-1.4a**						
**Males, OHIO, white**	**18.2**	**10.3**	**24.2**	**1970–2001**	**-1.4a**						
**Males, OHIO, black**	**22.6**	**-4.6**		**1970–2001**	**-1.6a**						
*Males, USA*	*17.2*	-		*1970–1975*	*-2.3a*	*1976–1993*	*-1.2a*	*1994–2001*	*-2.4a*		
*Males, USA, white*	*16.5*	-	**43.6**	*1970–1975*	*-2.6a*	*1976–1993*	*-1.3a*	*1994–2001*	*-2.1a*		
*Males, USA, black*	*23.7*	-		*1970–1984*	*-0.7a*	*1985–1994*	*-1.7a*	*1995–2001*	*-4.5a*		
											
**Females, OHIO**	**20.7**	**4.0**		**1970–2001**	**-1.5a**						
**Females, OHIO, white**	**20.2**	**6.9**	**29.2**	**1970–2001**	**-1.6a**						
**Females, OHIO, black**	**26.1**	**-7.8**		**1970–2001**	**-1.3a**						
*Females, USA*	*19.9*	-		*1970–1977*	*-2.2a*	*1978–1989*	*-1.1a*	*1990–2001*	*-1.7a*		
*Females, USA, white*	*18.9*	-	**49.7**	*1970–1980*	*-2.0a*	*1981–1983*	*0.0*	*1984–2001*	*-1.6a*		
*Females, USA, black*	*28.3*	-		*1970–1975*	*-3.4a*	*1976–1990*	*-0.7a*	*1991–2001*	*-1.9a*		
											
***Age 45–64***											
**Males, OHIO**	**274.3**	**5.5**		**1970–1985**	**0.3a**	**1986–2001**	**-1.9a**				
**Males, OHIO, white**	**263.9**	**6.2**	**53.7**	**1970–1984**	**0.2**	**1985–2001**	**-1.6a**				
**Males, OHIO, black**	**405.7**	**-0.7**		**1970–1985**	**0.8a**	**1986–2001**	**-2.8a**				
*Males, USA*	*259.9*	-		*1970–1977*	*0.4a*	*1978–1989*	*-0.2a*	*1990–2001*	*-2.3*		
*Males, USA, white*	*248.5*	-	**64.4**	*1970–1988*	*0.0*	*1989–2001*	*-2.1a*				
*Males, USA, black*	*408.6*	-		*1970–1977*	*1.7a*	*1978–1989*	*0.0*	*1990–2001*	*-3.0*		
											
**Females, OHIO**	**221.9**	**5.0**		**1970–1985**	**0.3a**	**1986–2001**	**-1.5a**				
**Females, OHIO, white**	**217.4**	**4.8**	**28.1**	**1970–1986**	**0.2**	**1987–2001**	**-1.5a**				
**Females, OHIO, black**	**278.4**	**1.2**		**1970–1984**	**0.9a**	**1985–2001**	**-2.0a**				
*Females, USA*	*211.3*	-		*1970–1986*	*0.1*	*1987–1992*	*-0.9a*	*1993–2001*	*-2.0a*		
*Females, USA, white*	*207.5*	-	**32.6**	*1970–1987*	*0.1*	*1988–1993*	*-1.2a*	*1994–2001*	*-2.0*		
*Females, USA, black*	*275.1*	-		*1970–1989*	*0.2a*	*1990–2001*	*-1.8*				
											
***Age 65+***											
**Males, OHIO**	**1,382.7**	**8.7**		**1970–1981**	**1.2a**	**1982–1993**	**0.2**	**1994–2001**	**-1.3a**		
**Males, OHIO, white**	**1,350.4**	**7.9**	**34.0**	**1970–1981**	**1.1a**	**1982–1993**	**0.2**	**1994–2001**	**-1.2a**		
**Males, OHIO, black**	**1,809.6**	**6.0**		**1970–1988**	**1.4a**	**1989–2001**	**-1.0a**				
*Males, USA*	*1,272.2*	-		*1970–1979*	*1.1a*	*1980–1991*	*0.5a*	*1992–2001*	*-1.3a*		
*Males, USA, white*	*1,251.7*	-	**36.4**	*1970–1979*	*1.0a*	*1980–1991*	*0.4a*	*1992–2001*	*-1.2a*		
*Males, USA, black*	*1,707.2*	-		*1970–1981*	*2.3a*	*1982–1988*	*1.7a*	*1989–1992*	*0.5*	*1993–2001*	*-1.9a*
											
**Females, OHIO**	**840.8**	**7.3**		**1970–1994**	**1.1a**	**1995–2001**	**-0.9a**				
**Females, OHIO, white**	**829.2**	**5.9**	**19.8**	**1970–1994**	**1.0a**	**1995–2001**	**-0.9a**				
**Females, OHIO, black**	**993.7**	**12.4**		**1970–1995**	**1.4a**	**1996–2001**	**-1.7a**				
*Females, USA*	*783.4*	-		*1970–1974*	*0.0*	*1975–1983*	*1.3a*	*1984–1992*	*1.0a*	*1993–2001*	*-0.4a*
*Females, USA, white*	*783.3*	-	**12.9**	*1970–1974*	*0.0*	*1975–1983*	*1.3a*	*1984–1992*	*0.9a*	*1993–2001*	*-0.4a*
*Females, USA, black*	*884.2*	-		*1970–1974*	*0.3*	*1975–1992*	*1.7a*	*1993–2001*	*-0.6a*		

(a) – p < 0.05

# – EAPC – Estimated Annual Percentage Change, ## – Age-adjusted standardized rate (World standard population)

**Table 2:** Cancer mortality in Ohio, 1997–2001, by gender and race

**Cancer site**	**Rate^#^**	**No of deaths**	**%**	**Cancer site**	**Rate^#^**	**No of deaths**	**%**
			
	**Male – White**				**Male – Black**		
*All sites*	*152.1*	*56,924*	*100*	*All sites*	*212.8*	*7,057*	*100*
							
Lung	52.3	18,996	33.4	Lung	70.9	2,306	32.7
Colon and Rectum	15.4	5,897	10.4	Prostate	31.1	1,130	16.0
Prostate	12.4	5,628	9.9	Colon and Rectum	21.0	705	10.0
Non-Hodgkin Lymphoma	7.0	2,642	4.6	Pancreas	10.0	328	4.6
Pancreas	7.0	2,569	4.5	Esophagus	7.7	233	3.3
Leukemia	6.3	2,302	4.0	Stomach	6.8	230	3.3
Esophagus	5.4	1,913	3.4	Liver	6.8	218	3.1
Urinary Bladder	4.4	1,810	3.2	Leukemia	6.1	203	2.9
Kidney	4.2	1,495	2.6	Myeloma	5.9	199	2.8
Brain	4.4	1,395	2.5	Non-Hodgkin Lymphoma	5.0	168	2.4

	**Female – White**				**Female – Black**		

*All sites*	*107.2*	*53,996*	*100*	*All sites*	*132.2*	*6,325*	*100*
							
Lung	28.1	13,274	24.6	Lung	33.3	1,524	24.1
Breast	18.2	8,520	15.8	Breast	25.3	1,134	17.9
Colon and Rectum	10.7	6,236	11.5	Colon and Rectum	14.5	759	12.0
Ovary	5.8	2,747	5.1	Pancreas	7.8	401	6.3
Pancreas	4.8	2,706	5.0	Ovary	4.0	195	3.1
Non-Hodgkin Lymphoma	4.5	2,541	4.7	Myeloma	3.6	191	3.0
Leukemia	3.7	1,977	3.7	Corpus and Uterus, NOS	3.7	179	2.8
Corpus and Uterus, NOS	2.7	1,379	2.6	Leukemia	3.8	178	2.8
Brain	2.8	1,093	2.0	Stomach	3.0	173	2.7
Kidney	2.0	1,008	1.9	Cervix Uteri	3.2	152	2.4

# – Age-adjusted mortality rate (World Standard Population)
